# The Effects of Psychological Interventions on Symptoms and Psychology of Functional Dyspepsia: A Systematic Review and Meta-Analysis

**DOI:** 10.3389/fpsyg.2022.827220

**Published:** 2022-04-08

**Authors:** Zhongcao Wei, Xin Xing, Xinxing Tantai, Cailan Xiao, Qian Yang, Xiaosa Jiang, Yujie Hao, Na Liu, Yan Wang, Jinhai Wang

**Affiliations:** ^1^Department of Gastroenterology, The Second Affiliated Hospital, Xi'an Jiaotong University, Xi'an, China; ^2^Department of Cardiology, The Second Affiliated Hospital, Xi'an Jiaotong University, Xi'an, China

**Keywords:** psychological interventions, functional dyspepsia, psychotherapy, meta-analysis, psychology

## Abstract

**Background:**

The effects of psychological interventions on symptoms and psychology of functional dyspepsia (FD) remain unclear. We aimed to comprehensively evaluate the effects of psychological interventions on symptoms and psychology of FD.

**Methods:**

We searched the PubMed, Cochrane Library, and Embase electronic databases for randomized controlled trials (RCTs) evaluating the role of psychological interventions in FD patients published before July 2021. Standardized mean differences (SMDs), risk ratios (RRs) and 95% confidence intervals (CIs) were calculated by a random effects model. Subgroup analyses and sensitivity analyses were also performed.

**Results:**

Fourteen RCTs with a total of 1,434 FD patients were included. Compared with the control group, psychological interventions were significantly more likely to symptom improvement [RR = 1.74, 95% CI (1.12, 2.72), *p* = 0.01], relieve gastrointestinal symptoms scores at follow up [SMD = −1.06, 95% CI (−1.55, −0.57), *p* < 0.0001], relieve gastrointestinal symptoms scores at end of treatment [SMD = −0.98, 95% CI (−1.29, −0.67), *p* < 0.001], decrease anxiety [SMD = −0.8, 95% CI (−1.38, −0.22), *p* = 0.006] and depression levels [SMD = −1.11, 95% CI (−1.62, −0.61), *p* < 0.001]. The results of the subgroup analysis showed that psychotherapy was more likely to symptom improvement, relieve gastrointestinal symptoms scores and decreased depression levels compared to the control.

**Conclusions:**

Psychological interventions may be effective in alleviating the symptoms and psychology of FD, but the effect appears to be limited to psychotherapy with fewer trials for other psychological interventions. More data from high-quality RCTs are needed to confirm their use in the treatment of FD.

## Introduction

Functional dyspepsia (FD) is a chronic gastrointestinal disease originating from the gastroduodenum without structural diseases (Wauters et al., 2020). FD is characterized by bothersome epigastric pain, epigastric burning, postprandial fullness or early satiety. FD is a highly prevalent disorder that affects ~5–20% of the world's population (Camilleri et al., 2005; Oshima and Miwa, 2015; Talley and Ford, 2016) and can significantly reduce the quality of life of patients, leading to increased medical costs. The pathophysiological mechanisms of FD are complex and may include impaired gastric regulation, delayed gastric emptying, excessive visceral sensitivity, low-grade mucosal inflammation, and eosinophilia in the duodenum (Masuy et al., 2019; Tziatzios et al., 2020). However, despite the continuous progress in research on the pathophysiological mechanism of FD, there are still no satisfactory methods for treating FD (Masuy et al., 2019).

At present, there is no available treatment that is effective for most patients with FD without significant side effects. The common treatment options for FD include Helicobacter pylori eradication, acid suppressive therapy, prokinetic agents, neuromodulators. Non-drug treatments such as acupuncture and psychological interventions may help to control symptoms, but there are still few relevant studies. FD is a heterogeneous disease in clinical symptoms and pathophysiology, which makes the development of effective treatment challenging (Masuy et al., 2019).

The brain gut axis is considered an important aetiological factor of FD (Drossman, 2016; Stanghellini et al., 2016). Psychological factors are an integral part of brain gut axis disorder, and psychological intervention may be a potential way to treat this complex disease (Masuy et al., 2019). A meta-analysis of psychological interventions on IBS showed that psychological interventions were effective for IBS treatment (Black et al., 2020). However, the effects of psychological interventions on symptoms and psychology of FD remain unclear. Previous systematic reviews of psychological interventions for FD patients have failed to draw firm conclusions due to the limited number of studies (Soo et al., 2011). A meta-analysis evaluating the effect of psychological interventions on FD showed that psychological interventions were effective in improving global FD symptom scores. However, since Cochrane Library databases were not retrieved, the number of included studies was limited, and only changes in gastrointestinal symptom scores were analyzed, changes in overall symptoms and psychology were not effectively analyzed (Rodrigues et al., 2021). At the same time, one of the research objects included in the meta-analysis was duodenal ulcer patients, which was not consistent with the diagnosis of FD and should be excluded (Wilhelmsen et al., 1994).

Hence, we performed a meta-analysis of randomized controlled trials to evaluate the effects of psychological interventions on symptoms and psychology of FD. Meanwhile, the therapeutic effect of different psychological intervention subtypes on FD patients was evaluated.

## Patients And Methods

### Search Strategy

The study was conducted in accordance with PRISMA guidelines. A literature search was conducted to assess the impact of psychological interventions on patients with FD. We searched the PubMed, Cochrane Library, and Embase electronic databases up to July 2021. In this meta-analysis, psychological interventions included psychotherapy, psychodrama, cognitive behavioral therapy, relaxation therapy and hypnosis. We searched the literature by using MeSH terms and free-text words. The MeSH terms included dyspepsia, psychotherapy, psychodrama, cognitive behavioral therapy, relaxation therapy and hypnosis (Soo et al., 2011). There was no language limitation. The PubMed search strategy was available in the Supplementary Method 1. In addition, we manually searched the reference lists of included manuscripts and reviews. The literature search was conducted independently by two researchers, and any inconsistencies in the search process were resolved by discussion with the third researcher.

### Inclusion Criteria

1. The study population was patients with FD (symptoms met one of the Rome I to Rome IV criteria).
2. The patients were 18 years or older.
3. The experimental group received psychological interventions (including psychotherapy, psychodrama, cognitive behavioral therapy, relaxation therapy and hypnosis). The control group received supportive therapy or no psychological intervention.
4. The outcomes included at least one measure of symptom relief, gastrointestinal symptoms scores, quality of life, psychological symptom, anxiety, or depression.
5. The study design was a randomized controlled trial (RCT).

### Exclusion Criteria

1. The patient had any type of organic gastrointestinal disease.
2. Heartburn and acid regurgitation were the main symptoms.

### Data Extraction

Data extraction was performed independently by two researchers. For each study, we collected the following data: name of the first author, article publication years, country of study population, study intervention, number of patients, age, symptom improvement, gastrointestinal symptom scores, quality of life, psychological symptom scores, anxiety, depression, follow-up time, and information needed for quality assessment. The gastrointestinal symptom scores were based on patients assessed symptoms. In the process of data extraction, any inconsistencies were resolved by consulting the third researcher.

### Quality Assessment

All the studies we included were RCTs, and the quality assessment was conducted by GRADEPro software. The quality of evidence was assessed by considering the following factors: risk of bias, inconsistency, indirectness, imprecision, publication bias, large effect, plausible confounding and dose-response gradient. The quality was rated as high, medium, low, or extremely low. Certainty of evidence was assessed for each outcome and risk of bias was assessed for each study. The risk of bias was assessed using the Cochrane Risk Bias Tool (ROB version 1.0). The key factors included the following: selection bias, performance bias, detection bias, attrition bias, reporting bias, and other biases. The quality assessment was conducted independently by two researchers, and any inconsistencies were resolved by consulting the third researcher.

### Statistical Analyses

Continuous variables were represented by the mean and standard deviation, and because the same outcome may have been evaluated differently, standardized mean differences (SMDs) and 95% confidence intervals (CIs) were used to evaluate continuous variables. The interpretation for this effect size: 0.2 represents a small effect, 0.5 a moderate effect, and 0.8 a large effect. For dichotomous variables, risk ratios (RRs) and 95% CIs were evaluated. The heterogeneity analysis was performed by Q statistic test, and we considered *p* < 0.1 and *I*^2^ > 50% to represent substantial heterogeneity. The meta-analysis was performed using a random effects model (DerSimonian–Laird method). Data were extracted from the intention-to-treat analysis of the original article. If the outcomes in the original article were shown as the median and interquartile range, the mean and SD values were calculated according to the method of Wan et al. (2014). In studies that only provided the standard deviation (SD) of pretreatment and post-treatment, the SD of the difference between pretreatment and post-reatment was calculated according to the following formula: SD^2^ = (SD _pretreatment_)^2^ + (SD _posttreatment_)^2^ − (2R ^*^ SD _pretreatment_^*^ SD _posttreatment_), and R was calculated from a included study reported in considerable detail (Cumpston et al., 2019). Sensitivity analysis was performed for outcomes assessed in ≥3 included studies by removing studies one by one to evaluate the impact of each study on the overall effect. The statistical analysis was mainly carried out by Review Manager 5.3 software. A *P* < 0.05 was considered statistically significant. According to the Cochrane Collaboration Handbook, testing for publication bias is not recommended as fewer than 10 studies were identified (Cumpston et al., 2019). Subgroup analysis was conducted to identify potential sources of heterogeneity. As psychological interventions included psychotherapy, psychodrama, cognitive behavioral therapy, relaxation therapy and hypnosis. Psychological intervention patterns were pre-defined variables that were considered in the subgroup analyses.

## Results

### Literature Search

**Figure 1:**
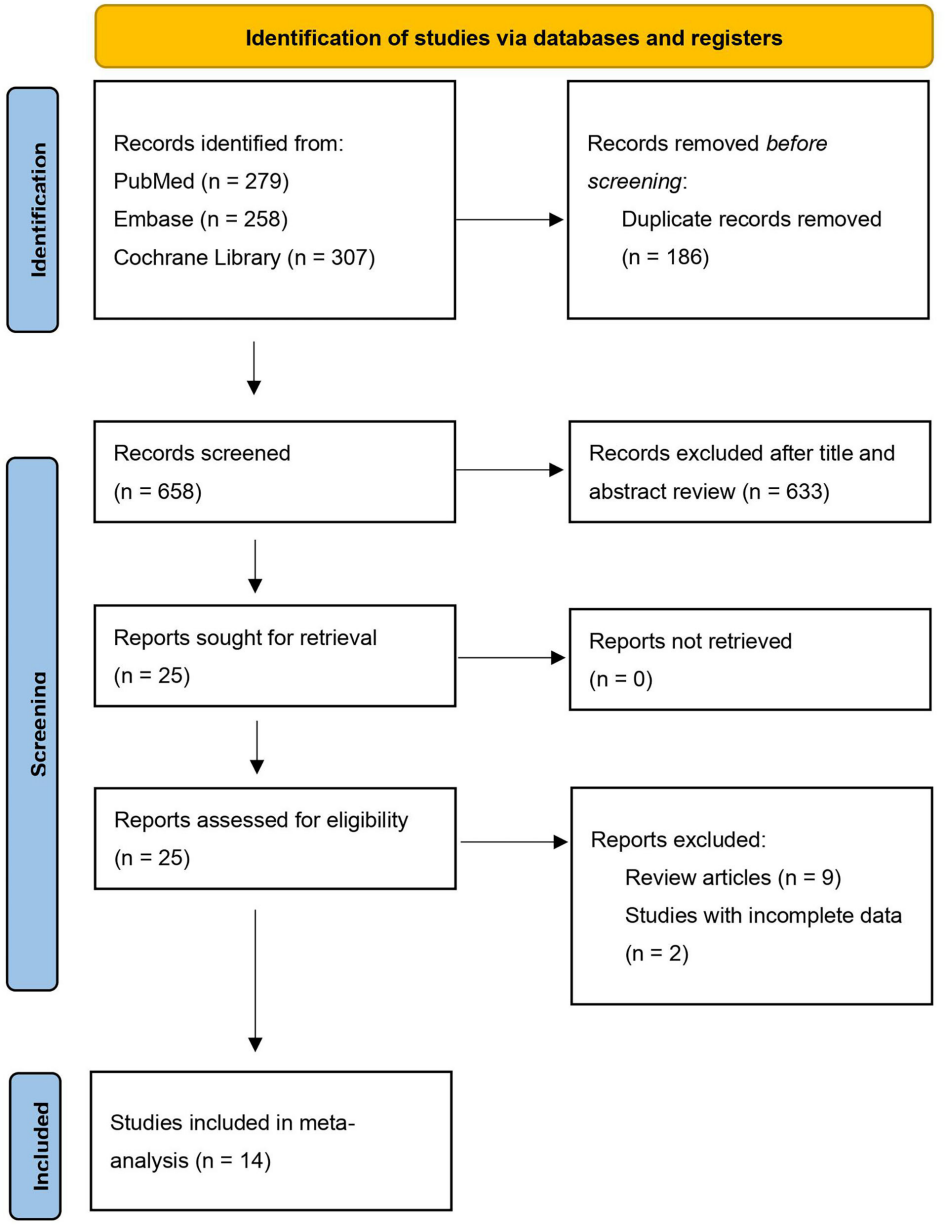
Flow chart of the literature search.

A total of 844 studies were found through the literature search, including 279 studies from PubMed, 307 from the Cochrane Library and 258 from the Embase database. After the elimination of 186 duplicate studies, the titles and abstracts of the remaining 658 studies were screened, and 633 studies were eliminated. In addition, 11 of the remaining studies did not meet the inclusion criteria: nine of them were review articles, 2 had incomplete data. Therefore, a total of 14 RCTs were included in our meta-analysis (Figure 1).

### Study Characteristics and Quality Assessment

**Figure 2:**
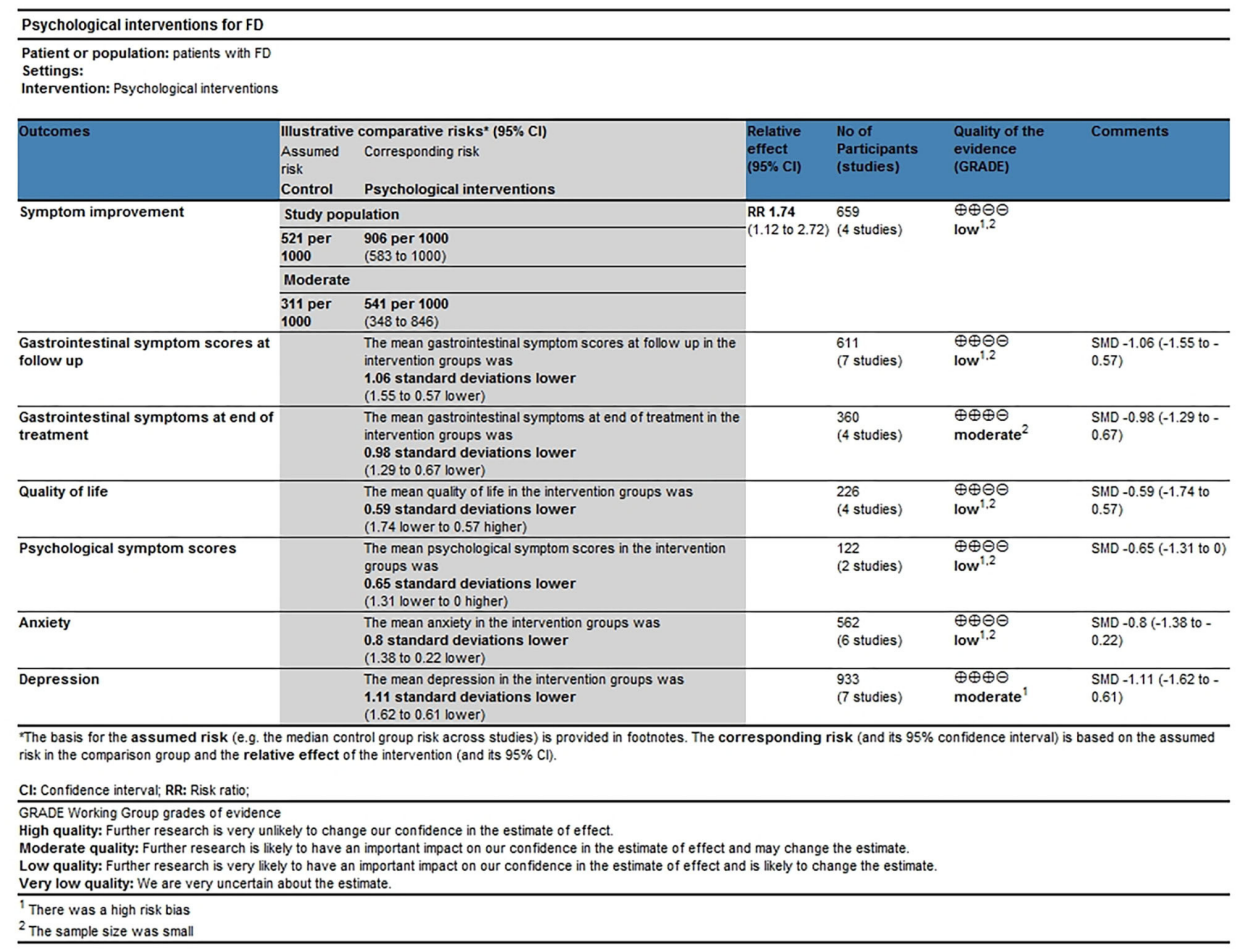
The GRADE quality assessment. FD, functional dyspepsia; SMDs, Standardized mean differences; RRs, risk ratios; CI, confidence interval.

The study characteristics are shown in Table 1. The included trials were conducted between 1988 and 2019, and two trials were conducted before 2000 (Bates et al., 1988; Haug et al., 1994). There were 14 RCTs (Bates et al., 1988; Haug et al., 1994; Hamilton et al., 2000; Calvert et al., 2002; Fan, 2006; Cheng et al., 2007; Haag et al., 2007; Hjelland et al., 2007; Jiang et al., 2008; Faramarzi et al., 2013; Dehghanizade et al., 2015; Orive et al., 2015; Zhuang, 2017; Xiong et al., 2019) with a total of 1434 FD patients (717 psychological interventions and 717 controls). There were 7 RCTs examining psychotherapy (Hamilton et al., 2000; Fan, 2006; Cheng et al., 2007; Hjelland et al., 2007; Jiang et al., 2008; Faramarzi et al., 2013; Orive et al., 2015), 1 RCT examining hypnotherapy (Calvert et al., 2002), 1 RCT examining relaxation or cognitive behavioral therapy (Haag et al., 2007), 3 RCT examining cognitive behavioral therapy (Haug et al., 1994; Dehghanizade et al., 2015; Xiong et al., 2019) and 2 RCT examining relaxation therapy (Bates et al., 1988; Zhuang, 2017). The number of participants in these RCTs ranged from 30 to 348. All studies provided the follow-up time, and the duration of the follow-up time ranged from 2 weeks to 14 months. The evidence level of the outcomes, i.e., symptom improvement, gastrointestinal symptoms scores, quality of life, psychological symptom scores, anxiety, and depression was assessed by GRADEPro software. The overall quality of evidence for all assessed outcomes was rated as low or moderate. The GRADE quality assessment is shown in Figure 2. The bias assessment of the included studies is shown in Supplementary Figures 1, 2.

**Table 1:** Characteristics of the included studies.

**References**	**Country**	**Study groups**	**No. of patients**	**Age (year)**	**Outcomes**	**Follow-up (month)**
Bates et al., 1988	Sweden	Psychosocial treatment (applied relaxation)	52	NA	J	13
		Control group	51	NA		
Haug et al., 1994	Norway	Cognitive psychotherapy (a form of CBT)	50	40	C	12
		Control group	50	40		
Hamilton et al., 2000	United Kingdom	Psychodynamic-interpersonal psychotherapy	31	40 ± 12	A, B, C, D, F	12
		Supportive therapy	27	40 ± 14		
Calvert et al., 2002	United Kingdom	Hypnotherapy	26	NA	A, C, D, E, G	14
		Supportive therapy	24	NA		
Fan, 2006	China	Regular gastric power medicine and repressing acid medicine + health education and psychologic support	51	18–72	H	1
		Regular gastric power medicine and repressing acid medicine	51	17–68		
Hjelland et al., 2007	Norway	Biofeedback group	20	36.8 ± 14.4	E	1
		Control group	20	33.8 ± 10.1		
Cheng et al., 2007	China	Flexible Coping Psychotherapy	33	18–65	C, G	12
		Supportive therapy	31	18–65		
Haag et al., 2007	Germany	Psychological interventions + intensive medical therapy	48	47.13 (39.4–53.6)	C, E, H	12
		Intensive medical therapy	28	44.4 (38.4–50.4)		
Jiang et al., 2008	China	Medicinal treatment + psychological intervention + life instruction	174	18–68	A, H	2
		Medicinal treatment	174	20–65		
Faramarzi et al., 2013	Iran	Brief psychoanalytic psychotherapy + medical treatment	20	31.92 ± 7.03	C, D, F, G, H	12
		Medical treatment	20	33.22 ± 5.29		
Orive et al., 2015	Spain	Medical therapy + psychotherapy	58	44.28 ± 14.06	A, B, C, D, G, H	6
		Medical therapy	70	47.09 ± 15.19		
Dehghanizade et al., 2015	Iran	Cognitive behavioral stress management	15	28.67 ± 7.09	E	1
		No intervention	15	28.67 ± 7.09		
Zhuang, 2017	China	Conventional nursing care + relaxation therapy	50	49.2 ± 10.3	G, H	0.5
		Conventional nursing care	50	47.6 ± 9.6		
Xiong et al., 2019	China	Comfort care (a form of CBT) and routine nursing	50	33.5 ± 4.1	C, G, H	2
		Routine nursing	50	32.5 ± 3.1		

*A, Symptom improvement; B, Health improvement; C, Gastrointestinal symptom scores at follow up; D, Gastrointestinal symptoms scores at end of treatment; E, Quality of life; F, Psychological symptom scores; G, Anxiety; H, Depression; J, pain intensity score; CBT, cognitive behavioral therapy*.

### Assessment of Heterogeneity

There is a certain degree of heterogeneity in our meta-analysis. The clinical heterogeneity was evidenced by different numbers of patients, a wide range of study locations, and different psychological interventions, and the length of follow-up was also different among the included studies (2 weeks to 14 months). The methodological heterogeneity was evidenced by different methods of measurement, although the studies we included were all RCTs. We used SMDs and 95% CIs to evaluate continuous variables because the same outcome may have been evaluated differently. We also performed a subgroup analysis to find the source of heterogeneity. We conducted a subgroup analysis of gastrointestinal symptoms scores, anxiety levels, and depression levels. After subgroup analysis, the heterogeneity between the psychotherapy group and other groups was not significantly reduced, indicating that the type of psychological intervention was not a source of heterogeneity. And considering the existence of heterogeneity, a random effects model was used.

### Psychological Interventions on Symptoms and Psychology

#### Psychological Interventions for Symptom Improvement

**Figure 3:**
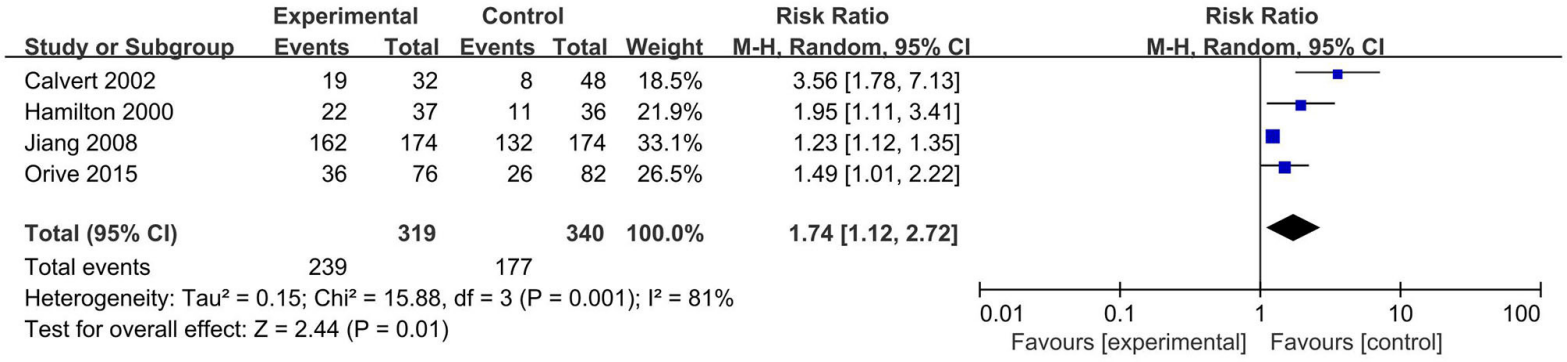
Meta-analysis of the effect of psychological interventions on symptom improvement. CI, confidence interval.

Four RCTs (Hamilton et al., 2000; Calvert et al., 2002; Jiang et al., 2008; Orive et al., 2015) evaluated the impact of psychological interventions on symptom improvement in 319 experimental groups and 340 control groups. Three RCTs (Hamilton et al., 2000; Jiang et al., 2008; Orive et al., 2015) used psychotherapy as psychological interventions, and one (Calvert et al., 2002) used hypnotherapy as psychological interventions. Symptom improvement was defined as symptoms were much or somewhat better than before. Hamilton et al. (2000) reported the number of people whose overall dyspeptic symptoms were better than before, Calvert et al. (2002) reported the number of people whose overall symptom score improved, Jiang et al. (2008) reported the number of people whose dyspeptic symptom score decreased by more than 80 or 50%, and Orive et al. (2015) reported the number of people with dyspepsia problem were much or somewhat better. The results of the meta-analysis showed that psychological interventions were more likely to symptom improvement than control group [RR = 1.74, 95% CI (1.12, 2.72), *p* = 0.01] (Figure 3). Heterogeneity analysis showed significant heterogeneity between studies (*p* = 0.001, *I*^2^ = 81%). In the subgroup analysis, compared with the control group, psychotherapy was more likely to symptom improvement [RR = 1.40, 95% CI (1.06, 1.86), *p* = 0.02], and heterogeneity was significantly reduced (*p* = 0.11, *I*^2^ = 54%) (Supplementary Figure 3).

#### Psychological Interventions for Gastrointestinal Symptoms Scores

**Figure 4:**
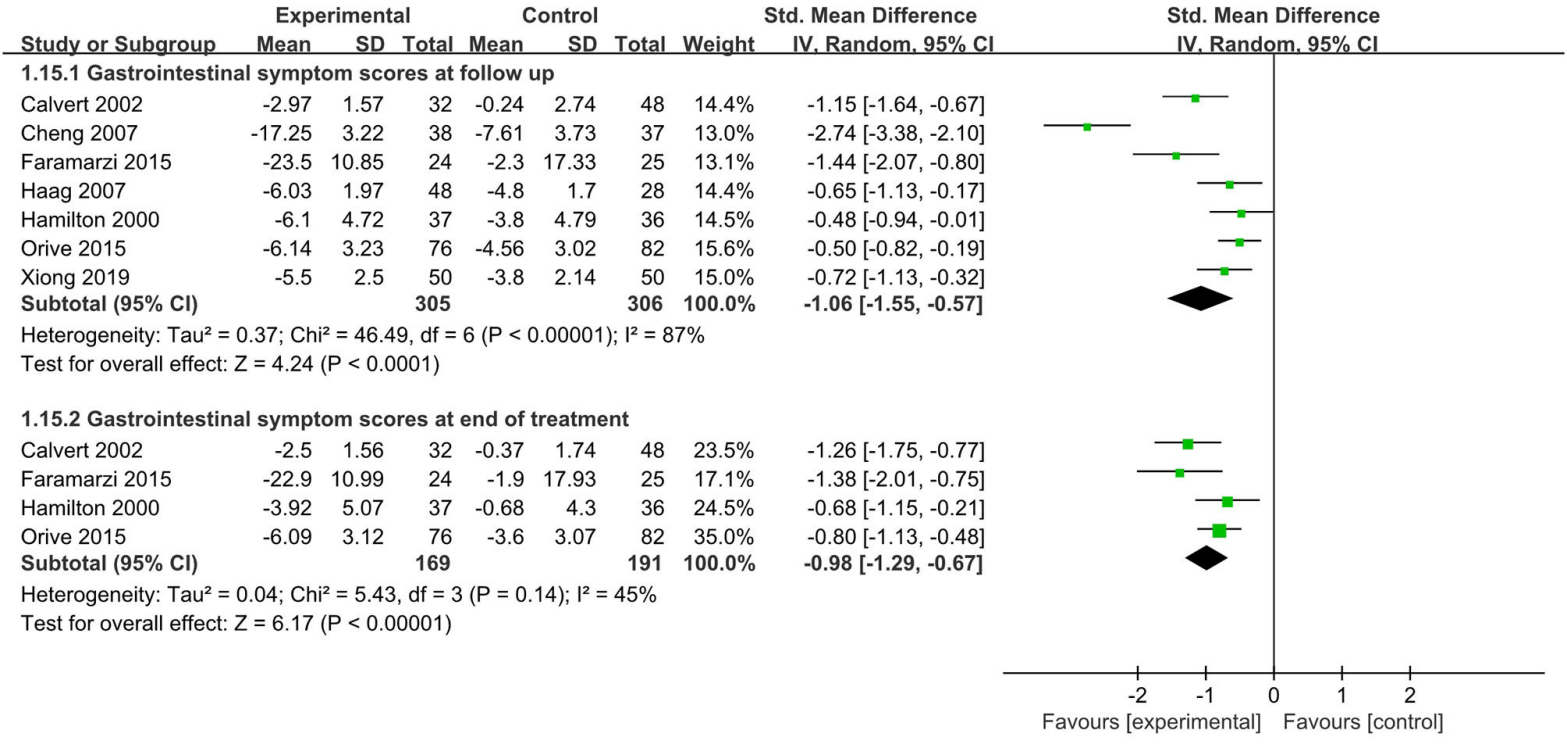
Meta-analysis of the effect of Psychological interventions on gastrointestinal symptom scores. CI, confidence interval.

A total of seven RCTs (Hamilton et al., 2000; Calvert et al., 2002; Cheng et al., 2007; Haag et al., 2007; Faramarzi et al., 2013; Orive et al., 2015; Xiong et al., 2019) with 611 patients (305 psychological interventions and 306 controls) reported a change in gastrointestinal symptoms scores at follow up. Four RCTs (Hamilton et al., 2000; Cheng et al., 2007; Faramarzi et al., 2013; Orive et al., 2015) used psychotherapy as psychological interventions, one (Calvert et al., 2002) used hypnotherapy as psychological interventions, one (Haag et al., 2007) used relaxation or cognitive behavioral therapy as psychological interventions, and one (Xiong et al., 2019) used cognitive behavioral therapy as psychological interventions. The gastrointestinal symptom scores were based on patients assessed symptoms. Hamilton et al. (2000) used 8 gastrointestinal symptoms scores, with a total score of 40; Calvert et al. (2002) used 6 gastrointestinal symptoms scores with a total score of 10; Cheng et al. (2007), 4 gastrointestinal symptoms were evaluated, with a total score of 40; Orive et al. (2015) evaluated 8 gastrointestinal symptoms, with a total score of 20; Haag et al. (2007) evaluated 5 gastrointestinal symptoms, with a total score of 45; Faramarzi et al. (2013) used 20 gastrointestinal symptoms to score, with a total score of 100, and Xiong et al. (2019) evaluated 8 gastrointestinal symptoms, with a total score of 48. Compared with the control, psychological interventions were more likely to reduce gastrointestinal symptoms scores at follow up, and there was a significant difference [SMD = - 1.06, 95% CI (- 1.55, - 0.57), *p* < 0.0001] (Figure 4). Heterogeneity analysis showed significant heterogeneity between studies (*p* < 0.001, *I*^2^ = 87%). The results of the subgroup analysis of psychological intervention types showed that there was a significant effect of psychotherapy on reducing gastrointestinal symptoms scores compared to the control [SMD = - 1.26, 95% CI (- 2.20, - 0.33), *p* = 0.008], and there was significant heterogeneity between studies (*p* < 0.001, *I*^2^ = 93%) (Supplementary Figure 4).

A total of four RCTs (Hamilton et al., 2000; Calvert et al., 2002; Faramarzi et al., 2013; Orive et al., 2015) with 360 patients (169 psychological interventions and 191 controls) reported a change in gastrointestinal symptoms scores at end of treatment. Three RCTs (Hamilton et al., 2000; Faramarzi et al., 2013; Orive et al., 2015) used psychotherapy as psychological interventions, and one (Calvert et al., 2002) used hypnotherapy as psychological interventions. Compared with the control, psychological interventions were more likely to reduce gastrointestinal symptoms scores at end of treatment [SMD = - 0.98, 95% CI (- 1.29 - 0.67), *p* < 0.001] (Figure 4). Heterogeneity analysis showed no significant heterogeneity between studies (*p* = 0.14, *I*^2^ = 45%).

#### Psychological Interventions on Quality of Life

**Figure 5:**
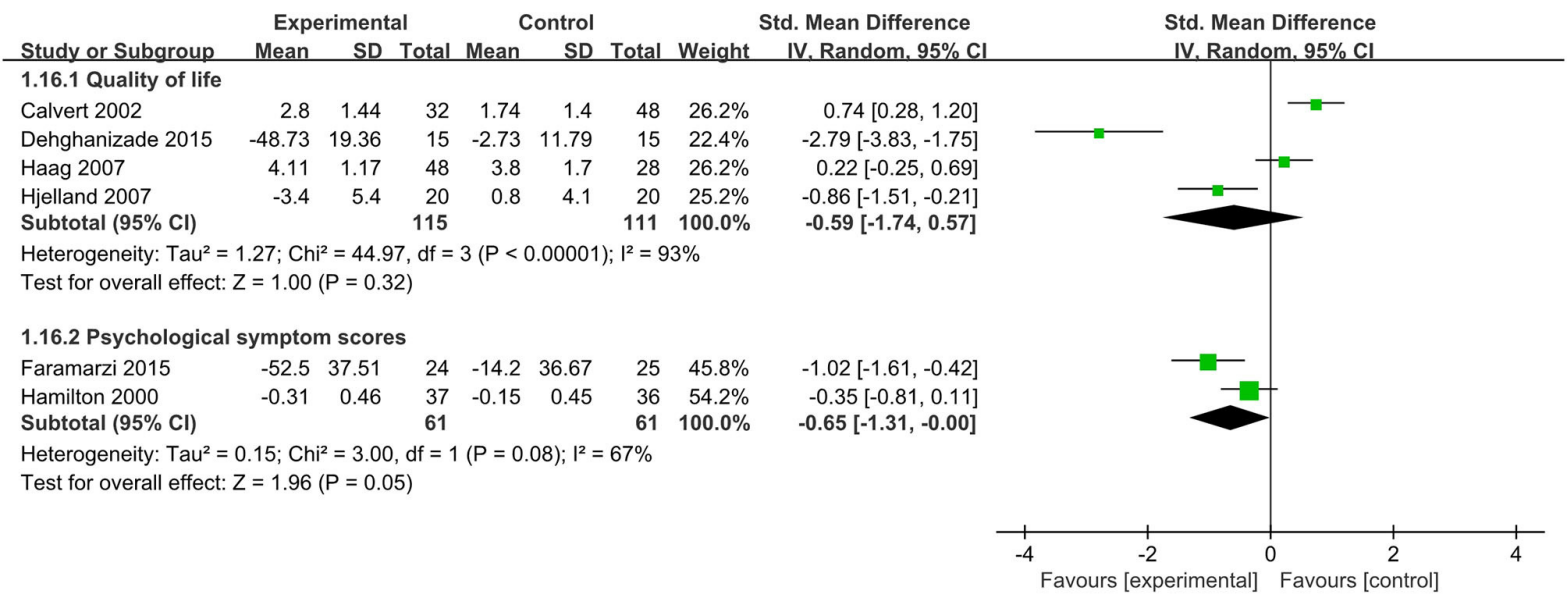
Meta-analysis of the effect of psychological interventions on quality of life and psychological symptom scores. CI, confidence interval.

Four RCTs (Calvert et al., 2002; Haag et al., 2007; Hjelland et al., 2007; Dehghanizade et al., 2015) reported the results of quality of life in 115 psychological interventions and 111 controls. Calvert et al. (2002) used hypnotherapy as psychological interventions and used a seven-item quality of life score of 10; Hjelland et al. (2007) used psychotherapy as psychological interventions, and assessed quality of life using the Short-form Nepean Dyspepsia Index, with a total score of 50. Haag et al. (2007) used relaxation or cognitive behavioral therapy as psychological interventions, and used the health-related quality-of-life, with a mean score of 50. Dehghanizade et al. (2015) used cognitive behavioral therapy as psychological interventions, and assessed quality of life using the Nepean Dyspepsia Index, with a total score of 100. Compared with the control, psychological interventions did not significantly improve the quality of life, and the difference was not statistically significant [SMD = −0.59, 95% CI (- 1.74, 0.57), *p* = 0.32] (Figure 5).

#### Psychological Interventions on Psychological Symptom Scores

Two RCTs (Hamilton et al., 2000; Faramarzi et al., 2013) evaluated the impact of psychological interventions on psychological symptom scores in 61 experimental groups and 61 control groups. Both RCTs used psychotherapy as psychological interventions, and psychological status was rated using the SCL-90-R. The results of the meta-analysis showed that psychological interventions were more likely to lower psychological symptom scores than control group; however, the significant difference was not critically significant [SMD = - 0.65, 95% CI (- 1.31, 0), *p* = 0.05] (Figure 5).

#### Psychological Interventions on Anxiety

**Figure 6:**
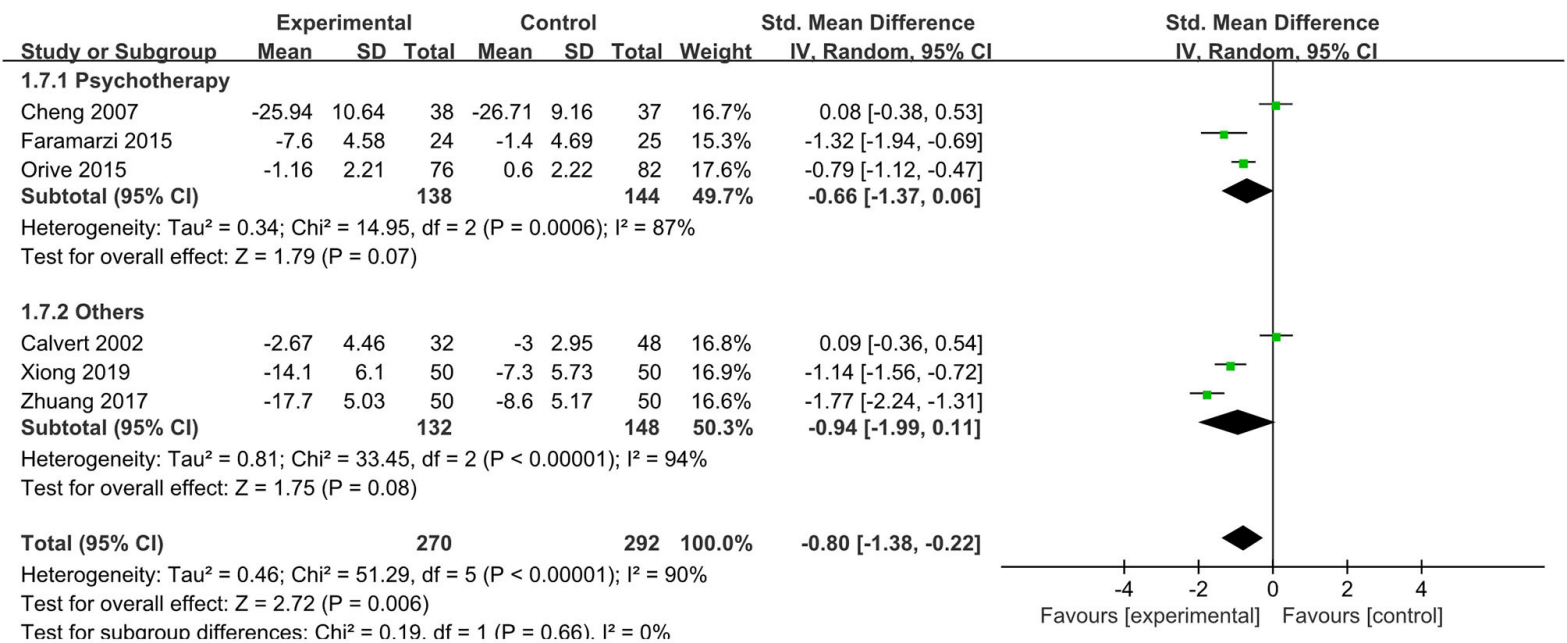
Meta-analysis of the effect of psychological interventions on anxiety. CI, confidence interval; Others, hypnotherapy/cognitive behavioral therapy/relaxation therapy.

Six studies (Calvert et al., 2002; Cheng et al., 2007; Faramarzi et al., 2013; Orive et al., 2015; Zhuang, 2017; Xiong et al., 2019) reported the data on anxiety levels (270 psychological interventions and 292 control). Calvert et al. (2002) examining hypnotherapy, three RCTs (Cheng et al., 2007; Faramarzi et al., 2013; Orive et al., 2015) used psychotherapy as psychological interventions, one RCT (Zhuang, 2017) used relaxation therapy as psychological interventions, and one RCT (Xiong et al., 2019) used cognitive behavioral therapy as psychological interventions. Calvert et al. (2002) and Orive et al. (2015) used the Hospital Anxiety Scale; Faramarzi et al. (2013) used an anxiety scale with a score ranging from 0 to 40, Cheng et al. (2007) used the State-Trait Anxiety Inventory to assess levels of anxiety, and the anxiety scores range from 20 to 80; Zhuang (2017) and Xiong et al. (2019) used the Self-rating Anxiety Scale with a cut-off score of 50. The analysis indicated that psychological interventions significantly reduce the anxiety levels compared with the control group (SMD = - 0.8, 95% CI: - 1.38,−0.22, *p* = 0.006) (Figure 6). Heterogeneity analysis showed significant heterogeneity between studies (*p* < 0.001, *I*^2^ = 90%). In the subgroup analysis, compared with the control group, psychotherapy was more likely to decrease anxiety levels, but the difference was not statistically significant [SMD = - 0.66, 95% CI (- 1.37, 0.06), *p* = 0.07]. Significant heterogeneity was observed in the subgroup analysis (*p* = 0.0006, *I*^2^ = 87%).

#### Psychological Interventions on Depression

**Figure 7:**
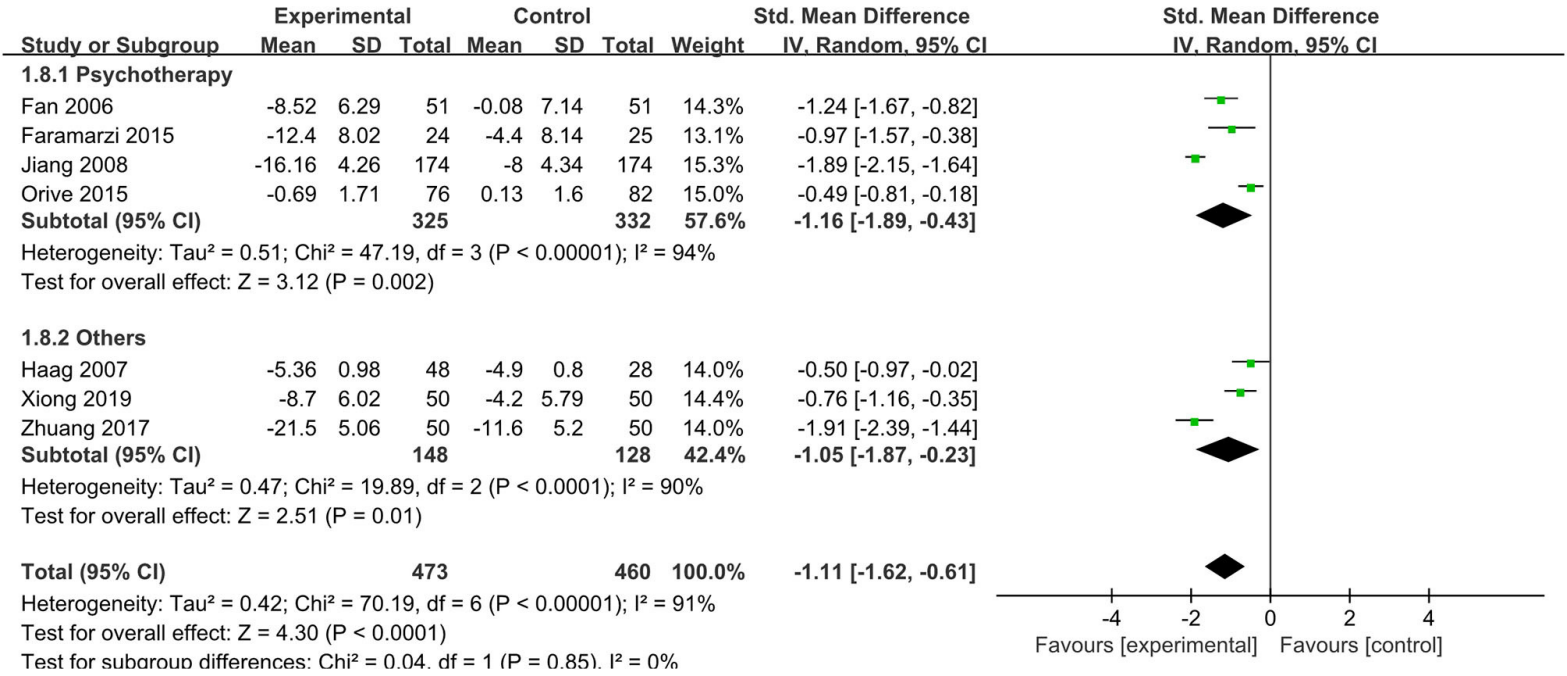
Meta-analysis of the effect of psychological interventions on depression. CI, confidence interval; Others, relaxation or cognitive behavioral therapy/cognitive behavioral therapy/relaxation therapy.

A total of seven RCTs (Fan, 2006; Haag et al., 2007; Jiang et al., 2008; Faramarzi et al., 2013; Orive et al., 2015; Zhuang, 2017; Xiong et al., 2019) with 933 patients (473 psychological interventions and 460 controls) reported a change in depression levels. Haag et al. (2007) examining relaxation or cognitive behavioral therapy, four RCTs (Fan, 2006; Jiang et al., 2008; Faramarzi et al., 2013; Orive et al., 2015) used psychotherapy as psychological interventions, one RCT (Zhuang, 2017) used relaxation therapy as psychological interventions, and one RCT (Xiong et al., 2019) used cognitive behavioral therapy as psychological interventions. Fan (2006) and Jiang et al. (2008) used the Hamilton Depression Scale (24 items) to assess the levels of anxiety; Haag et al. (2007) and Orive et al. (2015) used the Hospital depression Scale; Faramarzi et al. (2013) used a depression scale with a score ranging from 0 to 52, and Zhuang (2017) and Xiong et al. (2019) used the Self-rating Depression Scale with a cut-off score of 53. Compared with the control, psychological interventions were more likely to lower depression levels, and there was a significant difference [SMD = - 1.11, 95% CI (- 1.62, - 0.61), *p* < 0.0001] (Figure 7). The results of the subgroup analysis showed that there was a significant effect of psychotherapy on lowering depression levels compared to the control group [SMD = - 1.16, 95% CI (- 1.89, - 0.43), *p* = 0.002], and there was significant heterogeneity between studies (*p* < 0.001, *I*^2^ = 94%).

### Sensitivity Analysis

By removing studies one by one to evaluate the impact of each study, we found that there was a significant change in the heterogeneity of symptom improvement (heterogeneity *p* = 0.001, *I*^2^ = 81%). If Calvert's (Faramarzi et al., 2013) study or Jiang's (Faramarzi et al., 2013) study was removed, there was a significant reduction in heterogeneity (heterogeneity *p* = 0.11, *I*^2^ = 54%; heterogeneity *p* = 0.10, I^2^ = 56%). And there was a significant change in the heterogeneity of improving the quality of life (heterogeneity *p* = 0.0004, *I*^2^ = 87%). If Hjelland's (Faramarzi et al., 2013) study was removed, there was a significant reduction in heterogeneity (heterogeneity *p* = 0.12, *I*^2^ = 58%). Sensitivity analysis was also conducted on other outcomes that were reported in ≥3 included studies, and the heterogeneity was not significantly changed after removing studies one by one.

### Publication Bias

As the number of studies included for each outcome was <10, publication bias testing was not recommended according to the Cochrane Collaboration Handbook (Cumpston et al., 2019). It would be prudent to suspect that there were publication bias favoring positive outcomes. We cannot exclude the possibility that there are many more studies that could not prove a benefit of psychological interventions for FD.

## Discussion

To our knowledge, no meta-analysis has previously comprehensively assessed the effects of psychological interventions on symptoms and psychology of FD. The results of our meta-analysis showed that, compared with the control, there was a significantly beneficial effect of psychological interventions on symptom improvement, relieving gastrointestinal symptoms scores at follow up, relieving gastrointestinal symptoms scores at end of treatment, reducing anxiety and depression levels, with no significant improvement in the quality of life or psychological symptom scores. In the subgroup analysis, psychotherapy was more likely to symptom improvement, relieve gastrointestinal symptoms scores and decrease depression levels. These data thus lend support to psychological interventions as a therapeutic strategy that might be efficacious on symptoms and psychology of FD, but the effect appears to be limited to psychotherapy with fewer trials for other psychological interventions.

Population-based studies have shown that the incidence of psychological disorders in patients with FD is significantly higher than that in non-FD patients (Li et al., 2002; Castillo et al., 2004; Locke et al., 2004; Koloski et al., 2005; Gathaiya et al., 2009). Symptoms of FD were associated with psychiatric disorders, including anxiety and depression, but a causal relationship had not been established (Drossman et al., 1988). The pathophysiological studies of FD have shown that psychosocial factors may affect FD by regulating the processing and descending pathways of visceral signals in the brain (Van Oudenhove and Aziz, 2013). Previous meta-analyses of irritable bowel syndrome have shown that psychological interventions seem to be effective in the treatment of irritable bowel syndrome (Ford et al., 2014, 2019; Lee et al., 2014), and Ford et al. found that psychotropic drugs may be an effective treatment for FD (Ford et al., 2017). However, there are still few studies on the effect of psychological interventions on FD.

In this meta-analysis, psychological interventions included psychotherapy, psychodrama, cognitive behavioral therapy, relaxation therapy and hypnosis. Psychological interventions refer to any psychotherapeutic method designed to change a person's cognition, perception, or behavior (Shorey et al., 2021). Psychodynamic psychotherapy focuses on how maladaptive thoughts and behaviors occur (Soo et al., 2011). psychodynamic-interpersonal psychotherapy pays more attention to the relationship between therapist and patient. This method emphasizes that therapists and patients form a strong cooperative work alliance (Hamilton et al., 2000). The purpose of cognitive behavioral therapy is to improve the quality of life by changing patients' thoughts or thinking patterns and behaviors (Fordham et al., 2021). Relaxation therapy is to make patients experience the physical and mental pleasure brought by relaxation, so as to improve the psychological and physiological dysfunction caused by tension. Hypnotherapy is the use of hypnosis to treat patients in order to improve their condition. Although the effect appears to be limited to psychotherapy with fewer trials for other psychological interventions, the effect of other psychological interventions cannot be ignored. Calvert et al. found that hypnotherapy was highly effective in the management of FD (Calvert et al., 2002), and Haug et al. found that the cognitive psychotherapy group significantly reduced the symptoms of dyspepsia compared to the control group (Haug et al., 1994). Bates et al. found that pain intensity was significantly lower in the relaxation group than in the control group (Bates et al., 1988). Moreover, the studies included in our meta-analysis did not compare multiple psychological interventions, so it was not possible to compare the effects of various psychological interventions on functional dyspepsia. In clinical practice, the choice of psychological interventions needs to be comprehensively considered.

Previous reviews have assessed the effects of psychological interventions on FD. Popa et al. performed a review of 4 articles evaluating the efficacy of hypnotherapy in the treatment of FD (Popa et al., 2019). However, the patients in two of the articles were not explicitly diagnosed with FD. Soo et al. assessed the efficacy of psychological interventions on non-ulcerative dyspepsia but identified only four studies that failed to perform a meta-analysis and demonstrated insufficient evidence to confirm the efficacy of psychological intervention non-ulcerative dyspepsia (Soo et al., 2011). The results of Miller and Whorwell (2009) showed that hypnotherapy had considerable potential in the treatment of functional gastrointestinal disorders and should be included in the medical care of functional gastrointestinal disorders. Rodrigues et al. did not retrieve Cochrane Library databases, and only analyzed the changes in gastrointestinal symptom scores, without a comprehensive analysis (Rodrigues et al., 2021). Compared with the previous reviews, our meta-analysis included a total of 14 studies, which can provide a more comprehensive explanation of the effects of psychological interventions on symptoms and psychology of FD.

Our article had several limitations. First, there were differences in study populations, psychological intervention methods, and follow-up time among different included studies, which may affect the study results. We used SMDs and 95% CIs to evaluate continuous variables because the same outcome may have been evaluated differently. We also performed a subgroup analysis to find the source of heterogeneity. And considering the existence of heterogeneity, a random effects model was used. Second, although a total of 14 articles were included in our meta-analysis, a variety of psychological interventions were included, resulting in a small number of patients receiving each intervention. Third, our meta-analysis showed considerable clinical and methodological heterogeneity, and we cannot exclude publication bias in these very small numbers of studies; therefore, the efficacy of psychological interventions was likely to be overestimated.

In conclusion, Psychological interventions may be effective in alleviating the symptoms and psychology of FD, but the effect appears to be limited to psychotherapy with fewer trials for other psychological interventions. More data from high-quality RCTs are needed to confirm their use in the treatment of FD.

## Data Availability Statement

The original contributions presented in the study are included in the article/Supplementary Material, further inquiries can be directed to the corresponding author/s.

## Author Contributions

ZW, JW, and YW designed the study. XX, XT, and CX conducted the literature search and extracted the data. QY, XJ, YH, and ZW conducted the statistical analyses and drafted the manuscript. NL, YW, and JW contributed important intellectual content and critically revised the manuscript. All authors reviewed the final version and approved for submission.

## Funding

This work was supported by Key project of Shaanxi Province - social development (Grant No. 2018ZDXM-SF-046).

## Conflict of Interest

The authors declare that the research was conducted in the absence of any commercial or financial relationships that could be construed as a potential conflict of interest.

## Publisher's Note

All claims expressed in this article are solely those of the authors and do not necessarily represent those of their affiliated organizations, or those of the publisher, the editors and the reviewers. Any product that may be evaluated in this article, or claim that may be made by its manufacturer, is not guaranteed or endorsed by the publisher.
