# Supplementary material for: The Effects of Psychological Interventions on Symptoms and Psychology of Functional Dyspepsia: A Systematic Review and Meta-Analysis
Source: Front Psychol. 2022 Apr 8;13:827220. doi: 10.3389/fpsyg.2022.827220 (PMC9024246; doi:10.3389/fpsyg.2022.827220)
Supplement: Supplementary file 1 [file Table_1.DOCX]

**Supplementary Method 1** Search strategy for PubMed

1 "Dyspepsia"[MeSH Terms] OR "Dyspepsias"[Title/Abstract] OR "Indigestion"[Title/Abstract] OR "Indigestions"[Title/Abstract] OR "FD"[Title/Abstract] OR "functional dyspepsia"[Title/Abstract] OR "function dyspepsia"[Title/Abstract] OR "EPS"[Title/Abstract] OR "postprandial distress syndrome"[Title/Abstract] OR "PDS"[Title/Abstract] OR "epigastric pain syndrome"[Title/Abstract]

2 "Psychotherapy"[MeSH Terms] OR "Psychotherapies"[Title/Abstract] OR "Psychotherapists"[Title/Abstract] OR "Psychotherapist"[Title/Abstract] OR "clinical psychotherapists"[Title/Abstract] OR "clinical psychotherapist"[Title/Abstract] OR "schema therapy"[Title/Abstract] OR "schema therapies"[Title/Abstract] OR "Logotherapy"[Title/Abstract]

3 "Psychodrama"[MeSH Terms] OR "drama therapy"[Title/Abstract] OR "Dramatherapy"[Title/Abstract]

4 "Cognitive Behavioral Therapy"[MeSH Terms] OR "cognitive behavioral therapies"[Title/Abstract] OR "cognitive behavior therapy"[Title/Abstract] OR "cognitive therapy"[Title/Abstract] OR "cognitive behavior therapies"[Title/Abstract] OR "cognitive psychotherapy"[Title/Abstract] OR "cognitive psychotherapies"[Title/Abstract] OR "cognitive therapies"[Title/Abstract] OR "cognition therapy"[Title/Abstract] OR "cognition therapies"[Title/Abstract]

5 "Relaxation Therapy"[MeSH Terms] OR "relaxation techniques"[Title/Abstract] OR "relaxation technique"[Title/Abstract] OR "relaxation technics"[Title/Abstract] OR "relaxation technic"[Title/Abstract] OR "nature therapy"[Title/Abstract] OR "nature therapies"[Title/Abstract] OR "Ecotherapy"[Title/Abstract]

6 "Hypnosis"[MeSH Terms] OR "Hypnoses"[Title/Abstract] OR "Hypnotism"[Title/Abstract] OR "Hypnotherapy"[Title/Abstract] OR "Hypnotherapies"[Title/Abstract] OR "Mesmerism"[Title/Abstract]

7 2 OR 3 OR 4 OR 5 OR 6

8 1 AND 7


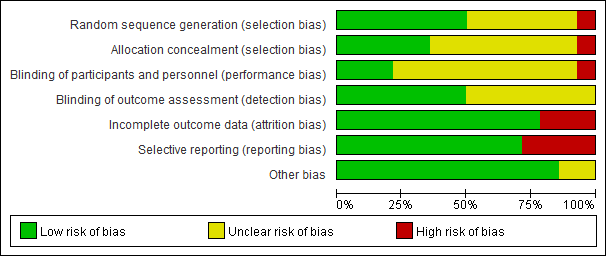


**Supplementary** **Fig. 1** Risk of bias graph.


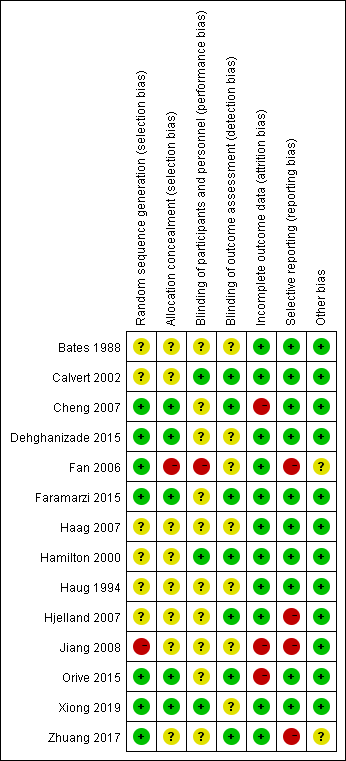


**Supplementary Fig. 2** Risk of bias summary.


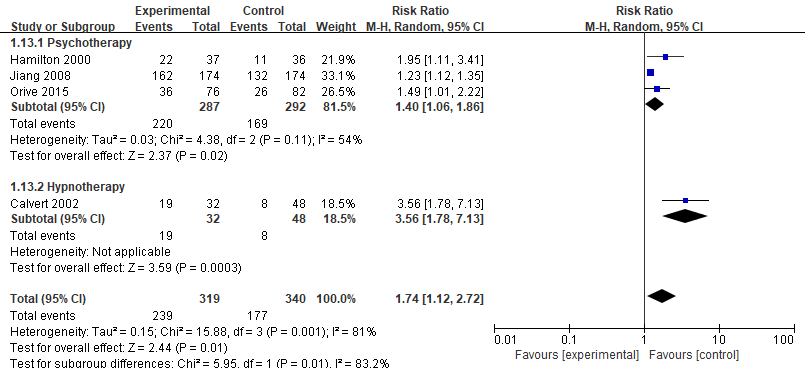


**Supplementary Fig. 3** Subgroup analysis of the effect of psychological interventions on symptom improvement.

CI: confidence interval.


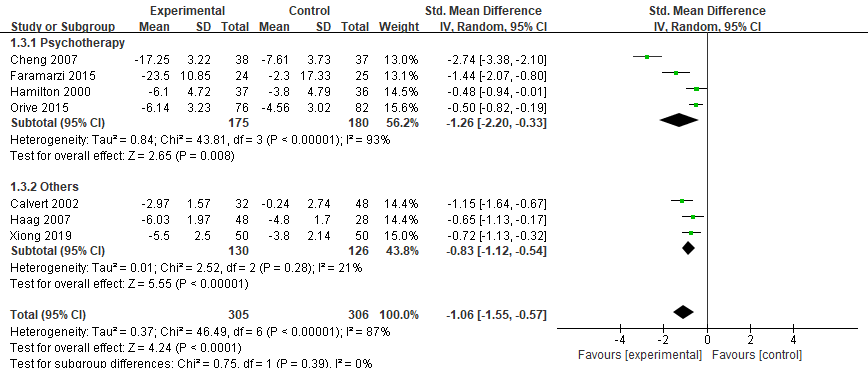


**Supplementary Fig. 4** Subgroup analysis of the effect of Psychological interventions on gastrointestinal symptom scores at follow up.

CI: confidence interval; Others: hypnotherapy/ relaxation or cognitive behavioural therapy/ cognitive behavioural therapy.
